# Environmental Effects on Viable Virus Transport and Resuspension in Ventilation Airflow

**DOI:** 10.3390/v14030616

**Published:** 2022-03-16

**Authors:** Tatiana A. Baig, Meiyi Zhang, Brooke L. Smith, Maria D. King

**Affiliations:** Aerosol Technology Laboratory, Biological & Agricultural Engineering Department, Texas A&M University, College Station, TX 77843, USA; tatianabaig18@tamu.edu (T.A.B.); teresa1217@tamu.edu (M.Z.); blsmith5@tamu.edu (B.L.S.)

**Keywords:** environmental factors, virus aerosols, PRD1 bacteriophage, bovine coronavirus (BCoV), wetted wall cyclone (WWC), nebulizer

## Abstract

To understand how SARS-CoV-2 spreads indoors, in this study bovine coronavirus was aerosolized as simulant into a plexiglass chamber with coupons of metal, wood and plastic surfaces. After aerosolization, chamber and coupon surfaces were swiped to quantify the virus concentrations using quantitative polymerase chain reaction (qPCR). Bio-layer interferometry showed stronger virus association on plastic and metal surfaces, however, higher dissociation from wood in 80% relative humidity. Virus aerosols were collected with the 100 L/min wetted wall cyclone and the 50 L/min MD8 air sampler and quantitated by qPCR. To monitor the effect of the ventilation on the virus movement, PRD1 bacteriophages as virus simulants were disseminated in a ¾ scale air-conditioned hospital test room with twelve PM2.5 samplers at 15 L/min. Higher virus concentrations were detected above the patient’s head and near the foot of the bed with the air inlet on the ceiling above, exhaust bottom left on the wall. Based on room layout, air measurements and bioaerosol collections computational flow models were created to visualize the movement of the virus in the room airflow. The addition of air curtain at the door minimized virus concentration while having the inlet and exhaust on the ceiling decreased overall aerosol concentration. Controlled laboratory experiments were conducted in a plexiglass chamber to gain more insight into the fundamental behavior of aerosolized SARS-CoV-2 and understand its fate and transport in the ambient environment of the hospital room.

## 1. Introduction

A novel human coronavirus, Severe Acute Respiratory Syndrome Coronavirus 2 (SARS-CoV-2) that emerged in Wuhan, China, in December 2019, has reached a pandemic level of global incidence [1]. The virus can enter the human body through the eyes, mouth, or nose and replicate itself after binding to receptors in the lung and other organs. Studies indicate the SARS-CoV-2 virus can remain viable and infectious suspended in aerosols for hours and on surfaces up to days, enabling efficient aerosol and fomite transmission of SARS-CoV-2 [2,3,4,5,6]. A recent study showed that 6 feet may not be sufficient to protect against coronaviruses, which may travel up in droplets up to 27 feet, but it was received with skepticism [7]. A single sneeze may emit 40,000 droplets with a geometric mean size of 360.1 µm exhaled immediately at the mouth [8]. Over 87% of particles exhaled by flu influenza patients were under 1 µm [9]. However, a similar percentage was reported for significantly larger (0.3–0.5 mm) particles exhaled by subjects infected with rhinovirus [10]. A computational model created by Vuorinen et al. [11] within a multi-institutional project shows that a cough from a person in one aisle in a grocery store spreads as a cloud of nanosized particles over the shelves into the next aisle. Similar open-source simulations can be found on the internet for cough aerosols spreading over aisles in an airplane. Earlier studies indicate that human movement in an airplane cabin increases the risk of infections by reducing the overall deposition and removal rate of the suspended aerosols [12].

Hospitals and clinics follow rigorous guidelines to maintain hygiene at 35–60% relative humidity (RH) and 21–24 °C temperature values (NFPA 99), with a mandated six air exchanges per hour (6 ACH). The role of ventilation in removing exhaled bioaerosols in buildings to prevent cross infections has been extensively studied after the severe acute respiratory syndrome coronavirus (SARS-CoV) outbreak in 2003 and can help inform mitigation strategies for the current SARS-CoV-2 pandemic [13]. Swabs taken from the air exhaust outlets in a hospital tested positive for SARS-CoV-2, suggesting that small virus-laden droplets may be transported by airflows and deposited on vents [4]. Due to the limitations of the sampler used in the study, the virus was not detected in the airflow. A scientific study of aerosol particles in a cruise ship’s heating, ventilation and air conditioning system found no detectable SARS-CoV-2 transmission, on surfaces or in the air [14]. However, a combined dose–response modeling study indicates the potential for airborne transmission [15]. Viable coronavirus (SARS-CoV-2) found on the Diamond Princess Cruise ship surfaces weeks after people were evacuated indicates that the virus survives for longer times on surfaces and forms biofilm-like structures, which may be influenced by environmental conditions [16]. Recent studies reveal that poor air quality and atmospheric pollution may be linked to the spread of the virus resulting in a greater number of COVID-19 cases in polluted areas [17].

Despite the increasing number of studies, there are still many unknowns about how SARS-CoV-2 spreads indoors and its infectability. The objective of this study was to gain more knowledge about the effect of environmental factors on the transport and viability of virus aerosols in the built environment based on computational airflow modeling and virus aerosol collection using the wetted wall cyclone samplers developed in the aerosol technology laboratory where this study was conducted.

As ventilation systems are practically ubiquitous in common workplaces, the effect of air properties on the infectivity and transport of aerosolized viruses is one of the most important subjects for study to aide in reducing the spread of infectious viral particles. This study is one of the first comprehensive studies on the impact of environmental conditions including temperature, relative humidity, and air velocity on the transport, and deposition of airborne viruses. The identification of effective environmental conditions and development of optimized ventilation designs could significantly reduce the entrainment and spread of viable infectious viruses in the air. The authors’ previous work indicates that a combined modeling and sampling approach can be used to mitigate transport of airborne infectious microorganisms in a ventilated facility [18]. Based on the airflow model and the bioaerosol movement, an optimal air intake/exhaust design can be selected that would result in the highest sanitation requirement (i.e., the least number of infectious agents) in the airflow. This study addresses the need for an optimal air intake/exhaust design combined with optimal environmental conditions to reduce the amount of SARS-CoV-2 viral particles in the air by integrating aerobiology with particle tracking and computational fluid modeling. Ultimately, this work allows for a better understanding of the behavior of virus size particles and a redesign for ventilation systems for reduced virus transmission at facilities, with a potential for application to any built environment.

The outcome of this study has the potential to protect public health through continuous monitoring of viral concentrations, with sufficient throughput to detect dynamic changes in concentration levels in room-size spaces. The potential for efficient detection of viable virus aerosols and mitigation of their spread was assessed by conducting controlled experimental studies to establish the pattern for aerosolization, deposition, and resuspension of SARS-CoV-2 simulant viruses at different environmental conditions and modeling the airflow pattern in a model hospital room to determine the effect of ventilation on the entrainment and spread of virus aerosols.

## 2. Materials and Methods

### 2.1. Media and Sample Preparation

The modified protocol by Bonilla et al. [19] was used for media and sample preparation. The collected PRD1 bacteriophage samples and the *Salmonella enterica* Serovar Typhimurium LT2 (RD1) host (courtesy of Carlos Gonzalez at the Department of Plant Pathology and Microbiology at Texas A&M University, College Station, TX, USA) were both pipetted into soft agar overlay and poured onto prepared Luria Bertani (LB) agar plates. This process was repeated for all the collected samples as well as stock solution dilutions, nebulizer solution dilution, and the test room exhaust filter sample. The plates were placed in the 37 °C incubator over night to grow and the plaque forming units were counted the next day.

To prepare the Luria Bertani (LB) culture medium, 10 g of peptone, 5 g of yeast extract, and 5 g of NaCl were added per liter of MQ water and autoclaved. All media compounds were from Becton, Dickinson and Company (BD, Franklin Lakes, NJ, USA). The soft agar medium was prepared by adding 25 g of LB broth powder and 7.5 g of agar per liter of MQ water. The soft agar was prepared in 50 mL batches and autoclaved.

The bacteriophage host *Salmonella enterica* Serovar Typhimurium LT2 (RD1) bacterium was grown in LB culture medium containing 100 mg/L Ampicillin, added after autoclaving, to select for cells with pRD1 as the PRD1 phage infects cells that harbor a conjugative plasmid [20]. The SM (Sodium-Magnesium) phage dilutant buffer for phage isolation and propagation with a pH of 7.4 was prepared by adding 5.8 g of NaCl, 2.0 g of MgSO_4_·7 H_2_O, and 50 mL of 1 M Tris-HCl per 1 L of MQ water, autoclaved, 0.2 μm filter sterilized and stored at room temperature. Calcium chloride (CaCl_2_) solution was prepared as a filter-sterilized 1 M stock solution and added to the LB broth to a final concentration of 0.001 M. The overlay was prepared by adding 0.1 mL of fresh mid-log phase (OD_600_ = 1.0) *S. enterica* host culture incubated at 37 °C, and 0.1 mL of phage (10^2^–10^3^ plaque forming units (PFU)/mL) to 4 mL of soft agar containing 100 mg/L Ampicillin at 56 °C in a 5 mL tube, poured over an LB agar plate and incubated overnight at 37 °C.

#### Phage Isolation and Propagation via Plate Lysate

After incubating the LB agar plate with the bacteria to form plaques at 37 °C, the plate was lysed by pouring 5 mL of SM buffer on top of the plate and shaking, gently, for 15 min. The phage suspension in the buffer was collected and centrifuged at 4000× *g* for 5 min to remove cell debris. The phage lysate was filter sterilized and stored at 4 °C.

### 2.2. Plexiglass Model Chamber Test

In a controlled plexiglass model chamber at Biosafety Level 2, a Collison 6-jet nebulizer (BGI, Waltham, MA, USA) was used to disseminate known numbers of bovine coronavirus (BCoV) aerosols. The laboratory plexiglass chamber experiments were used to simulate SARS-CoV-2 in controlled environmental conditions and mitigate uncertainty of testing in the open environment of the hospital model room. BCoV (bovine coronavirus, Mebus, Catalog No. NR-445, https://www.beiresources.org/, accessed on 11 March 2022), an enveloped, positive-sense, single-stranded RNA betacoronavirus, served as the simulant virus for the SARS-CoV-2 coronavirus throughout this study which allowed for the generalization of results to SARS-CoV-2. A series of experiments were performed at different temperatures and relative humidity values to assess the effect of environmental factors on particle deposition. At regular time intervals after aerosolization, the cabinet surfaces were swiped to quantify the number of impacted viral particles using quantitative polymerase chain reaction (qPCR). The total virus concentrations that became resuspended in the air were collected with the wetted wall cyclone (WWC) bioaerosol collectors including stainless steel and 3D printed cyclone units and the MD8 Airport Sampler (Sartorius, Goettingen, Germany) as reference. The WWC collected bioaerosols at 100 L/min which continuously collected particles into MQ water by concentrating virus particles from the air in the cyclone into the exhaust in the MQ water. The virus samples were quantitated with qPCR.

#### 2.2.1. Plexiglass Model Chamber Set-Up

A controlled temperature–humidity BSL-2 chamber (0.91 m × 0.91 m × 0.61 m) was built of clear Plexi-glass Polypropylene with 3 mm thickness. A Collison 6-jet nebulizer (BGI, Waltham, MA, USA) was used to disseminate known numbers of BCoV aerosols at different relative humidity (30, 60 and 80%) and temperature (10, 15, 25 °C) values monitored by probes. The range of temperature and relative humidity values was based on the measurements the authors conducted in meat processing facilities in preliminary studies. A fan with a HEPA filter (high-efficiency particulate air (filter); to provide particle free background air) and a flow straightener were connected to the inlet to maintain the airflow at approximately 0.1 m/s in the chamber, corresponding to the average indoor air velocity [21]. An additional HEPA filter was inserted at the chamber exit. HOBO^®^ dataloggers (Onset, Bourne, MA, USA) and hot wire anemometer (TSI Inc., Shoreview, MN, USA) were used for temperature, relative humidity, and air velocity measurements. HOBO dataloggers are wireless loggers with built-in sensors that record temperature and relative humidity of the surrounding environment during its operation period, in which data can be downloaded through HOBOware program for further analysis. Hot wire anemometers measure air velocity through heat transfer from an electrically heated wire and display the reading on its integrated screen in real time.

The number of viral particles impacted on the walls and metal, plastic, and wood surface samples (2 in. × 1 in.) at the bottom of the chamber were quantitated to assess the effect of temperature and humidity on particle deposition. The virus aerosols that remained resuspended in the chamber were collected using the WWC, the 3D printed WWC, both operated at 100 L/min, and the MD8 Sartorius Airport Sampler, operated at 50 L/min, using 80 mm gelatin filters as reference. Each test was performed three times.

#### 2.2.2. Virus Quantitation by qPCR

For total viral counts for all samples, whole cell qPCR (quantitative Polymerase Chain Reaction) was performed using BCoV specific oligonucleotides and probes [22,23]. The BCoV sample was added to the qPCR SYBR Green reaction mixture (Applied Biosystems, Warrington, UK), and amplified in an automated thermocycler/analyzer (AB StepOne RT-PCR System, AB, Foster City, CA, USA) as described previously [24]. Broadly reactive primer pairs IN-2 (+) 5′ GGGTTGGGACTATCCTAAGTGTGA 3′ and IN-4 (–) 5′ TAACACACAACICCATCATCA 3′ were used to identify the bovine coronavirus (GenBank Access NC_003045). Dilutions of a plasmid containing the BCoV target sequence were used to create a standard calibration curve for each analysis and allow for the calculation of total viral gene copy number [25].

#### 2.2.3. Surface Virus Assessment

**Figure 1:**
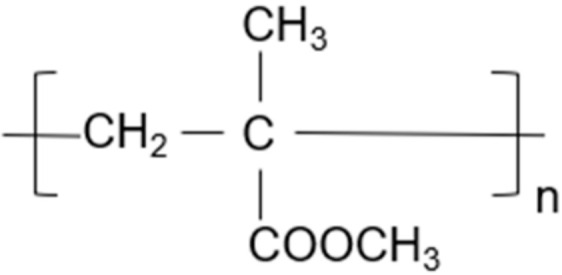
Chemical compound of polymethyl methacrylate (chamber surface).

**Figure 2:**
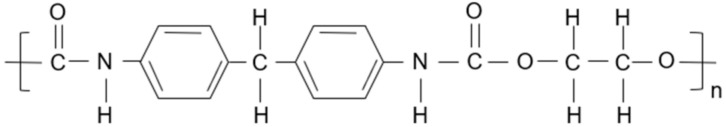
Chemical compound of polyurethane (wood surface).

**Figure 3:**
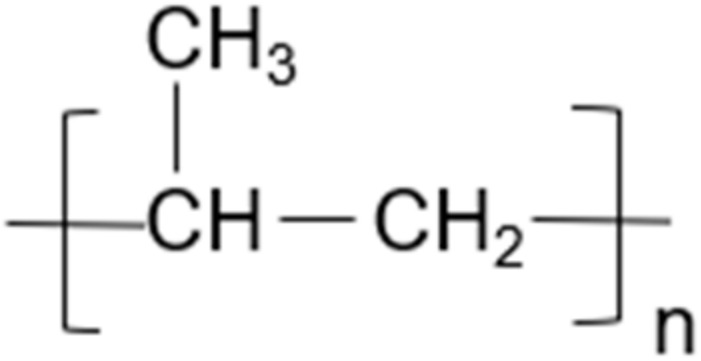
Chemical compound of polypropylene (plastic surface).

Three different surfaces were placed inside the chamber: wood, metal, and plastic that are commonly found, high-touch surfaces in hospital rooms. Figure 1 shows the chemical compound of the plexiglass chamber, polymethyl methacrylate (PMMA) [26]. The wood surface has a varnish finish made of polyurethane (PU), Figure 2 [27]. The metal surface used was aluminum. Polypropylene, (PP), its chemical compound shown in Figure 3 [28], was the plastic surface used in the chamber.

Bio-layer interferometry (BLI) was performed on the surface swab samples to determine the kinetics of the samples collected for each surface under the different environmental conditions. BLI is a technique used to measure the micromolar interactions through white light interference caused by the sample attaching to the biosensor surface. The BLItz system (ForteBio, Fremont, CA, USA) was used for the testing with Aminopropylsilane (APS) biosensors (ForteBio, Fremont, CA, USA). It is important to understand the hydrophobic interaction between BCoV and the hydrophobic APS biosensor because the S protein of SARS-CoV-2 binds to the angiotensin-converting enzyme 2 (ACE2) receptors of its host cells. This binding interaction occurs in a hydrophobic region. The BLI results will show how the S protein would bind to human cell receptors after coming into contact with these 3 surfaces in a hospital [29,30]. Each biosensor was hydrated in Phosphate Buffer Saline (PBS) buffer (pH 7.4) for 10 min before use. The tests had 3 steps, baseline, association, and dissociation. The baseline step was performed with 4 μL of PBS buffer for 30 s. The association step used 4 μL of sample for 300 s to allow the sample to associate to the surface and measure the association of the sample to the biosensor surface. Dissociation took 300 s with 4 μL of PBS buffer to allow the sample to dissociate from the surface and measure the sample dissociation from the biosensor surface. The BLItz system was used to generate binding curves with local modeling using a designated reference. Based on the curve, the association and dissociation rates and kinetics constants were determined for each surface swab sample.

### 2.3. Model Hospital Room Test

#### 2.3.1. Model Hospital Room and Equipment Setup

**Figure 4:**
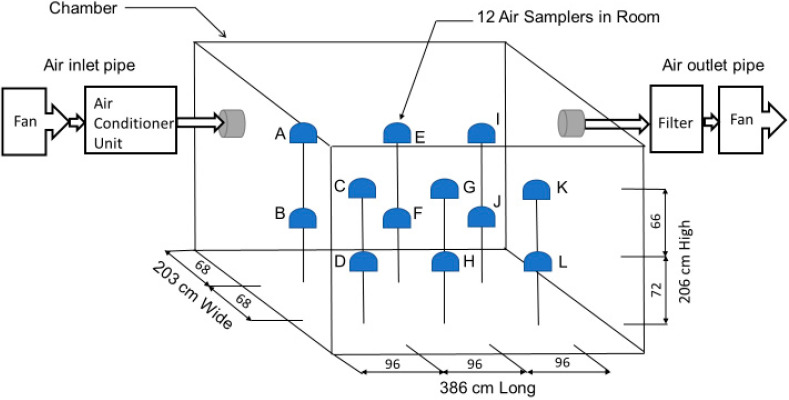
Hospital model room dimensions and locations of the PM2.5 samplers. Samplers A, C, E, G, I, and K are all upper samplers 136 cm above the ground and 66 cm above the lower samplers. The lower samplers B, D, F, H, J, and L are 72 cm above the ground.

**Figure 5:**
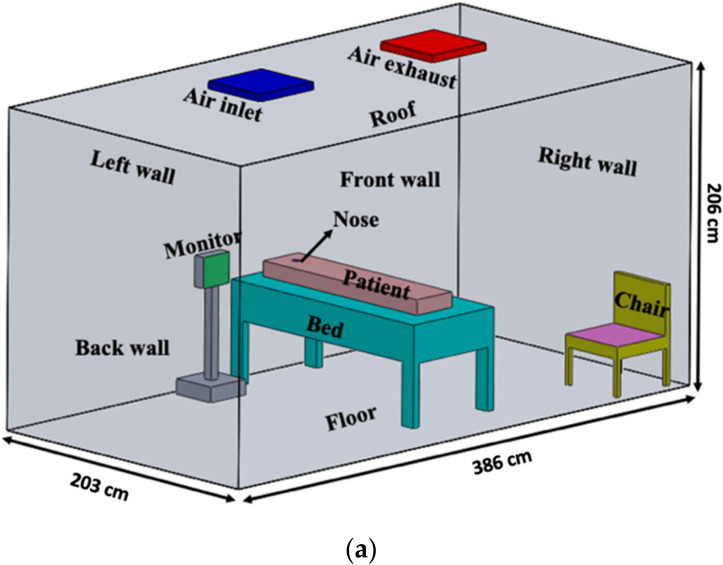

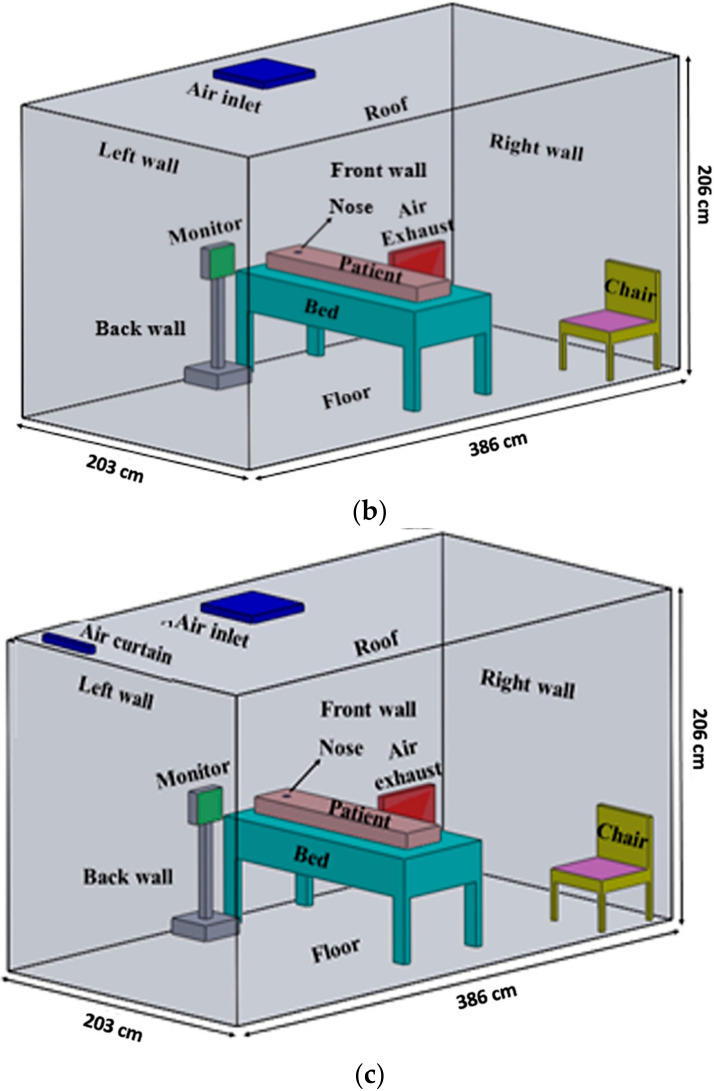
Hospital model room configuration (**a**) with the air inlet in the ceiling on the left, exhaust on the right; (**b**) with the air inlet in the ceiling above the bed, exhaust at the bottom left on the wall; and (**c**) with the air inlet in the ceiling above the bed, exhaust at the bottom left on the wall, air curtain entry/door. The schematics are showing the dimensions of the room and the positions of the objects in the room.

The Texas A&M University Biosafety Level 2, air-conditioned 16.14 m^3^, ¾ scale physical particle test room operated at an air exchange rate of 6 air changes per hour (ACH) was utilized to test PRD1 phages as SARS-CoV-2 simulants. Twelve PM2.5 filter samplers at 15 L/min air flow were used to collect the aerosolized virus (Figure 4 and Figure 5) [31]. Tests were conducted using three different ventilation configurations: (a) with the air inlet in the ceiling on the left, exhaust on the right; (b) with the air inlet in the ceiling above the bed, exhaust at the bottom left on the wall; and (c) with the air inlet in the ceiling above the bed, exhaust at the bottom left on the wall, air curtain entry/door (Figure 5).

During sampling, the ventilation system was operated constantly for each configuration and the environmental conditions (temperature, relative humidity, air velocity) were monitored. The virus particles were injected into the chamber with a Collison 24-jet atomizer at a location where the head of the patient lying on the bed in the room would be typically located. A 50 mL aliquot of the phage sample was added to 300 mL of SM buffer and nebulized as a SARS-CoV-2 simulant for 5 min aerosolization periods, resulting in a concentration of approximately 3.16 × 10^9^ PFU/m^3^ in the hospital room. The particle size distribution and mass concentration of the virus aerosols generated by the Collison nebulizer at 30.48 cm (head of the bed), 60.96 cm (middle of the bed), and 91.44 cm (foot of the bed) distance from the nebulizer were measured with the Aerodynamic Particle Sizer (APS, Model 3321, TSI Inc., Burnsville, MN, USA) at a sampling flow rate of 1 L/min. Twelve PM2.5 samplers with HTTP 0.4 µm membrane filters (Whatman, Nucleopore, GE Healthcare, Buckinghamshire, Amersham, UK) were placed in the model room to sample air at 15 L/min inflow rate. The HEPA filter at the air exhaust was also collected and analyzed after each test. Sampling was conducted at room temperature during the entire period of 5 min nebulization. Each test was repeated 3 times.

#### 2.3.2. Sample Assessment

For preliminary analysis of viable virus collection, each of the 12 filters collected in each test was placed in 2 mL of PBS. Aliquots of 4 mL of soft agar medium were added to glass tubes with 100 µg/mL Ampicillin. Aliquots of 100 μL of each collected sample in 10x dilution and 100 μL of the fresh mid-log phase *S. enterica* LT2 RD1 host were added to the medium and poured over a plate. A 10^−6^ dilution of either the nebulized liquid or the stock solution 100 μL was added to the soft agar medium with 100 μL of the *Salmonella* host and poured over an LB plate. The same process was performed with the resuspended exhaust filter. The plates were placed in the incubator overnight at 37 °C and the plaque forming units (PFUs) were counted the next morning. Total PRD1 phage numbers were quantitated by qPCR using species specific primers and ABI qPCR chemistry as described in Section 2.2.

#### 2.3.3. Computational Fluid Dynamics (CFD) Modeling and Validation

HOBOware dataloggers were used for the continuous recording of temperature and relative humidity data at the bioaerosol collection sites. The airflow was measured within every cubic meter of the chamber by anemometry to validate the flow model [32,33]. The HOBO units were connected to the 12 PM 2.5 samplers and started when the testing began. The data from the HOBOware Pro 3.7.22 were collected, exported, and analyzed in Excel worksheets. The SolidWorks^®^ 2020 SP5 program was used to create a model of the hospital model room, bed, visitor chair, and monitor based on the dimensions provided by the mechanical blueprint of the chamber. A patient was modeled as a 51.75 inch-long and 9.67 inch-wide rectangular prism based off the average height and waist circumference of a male in the U.S. [34]. The model was imported into ANSYS^®^ Fluent 2020 R2 software to create a detailed mesh. Each model and mesh were adjusted based on the ventilation configuration being modeled. The mesh sized used for each simulation was 700,000 elements. The standard k-ω turbulence model based on the Navier–Stokes equations was used to model unsteady room ventilation in steady flow conditions. Airflow through the space was simulated to determine virus movement in the room. For CFD simulation, in all ventilation configurations, the vertical walls, floor, monitor surface, hospital bed, chair, and air deflecting cones of the air diffuser were considered adiabatic. The transport equations, Equations (1) and (2), used by ANSYS are shown below [35].

Equation (1). Turbulence kinetic energy (*k*)

(1) $$\frac{\partial }{\partial t}\left(\rho k\right)+\frac{\partial }{\partial x_{i}}\left(\rho ku_{i}\right)=\frac{\partial }{\partial x_{j}}\left(\Gamma_{k}\frac{\partial k}{\partial x_{i}}\right)+G_{k}-Y_{k}+S_{k}$$

Equation (2). Specific dissipation rate (*ω*)

(2) $$\frac{\partial }{\partial t}\left(\rho \omega \right)+\frac{\partial }{\partial x_{i}}\left(\rho \omega u_{i}\right)=\frac{\partial }{\partial x_{j}}\left(\Gamma_{\omega }\frac{\partial \omega }{\partial x_{j}}\right)+G_{\omega }-Y_{\omega }+S_{\omega }$$

The initial conditions used for the model are shown in Table 1. Velocity and temperature conditions were based on measurements taken during experimental testing. The air exchange rate for all models was 6 air changes per hour (ACH) and the door was closed in all configurations.

### 2.4. Statistical Analysis

Statistical analysis was performed with Microsoft Excel (version 6.54) on all experimental data using ANOVA with a 95% confidence interval to determine if there was significant difference between replicates and experimental conditions for the plexiglass chamber and between replicates and ventilation configurations for the hospital model room.

## 3. Results

### 3.1. Plexiglass Model Chamber

**Figure 6:**
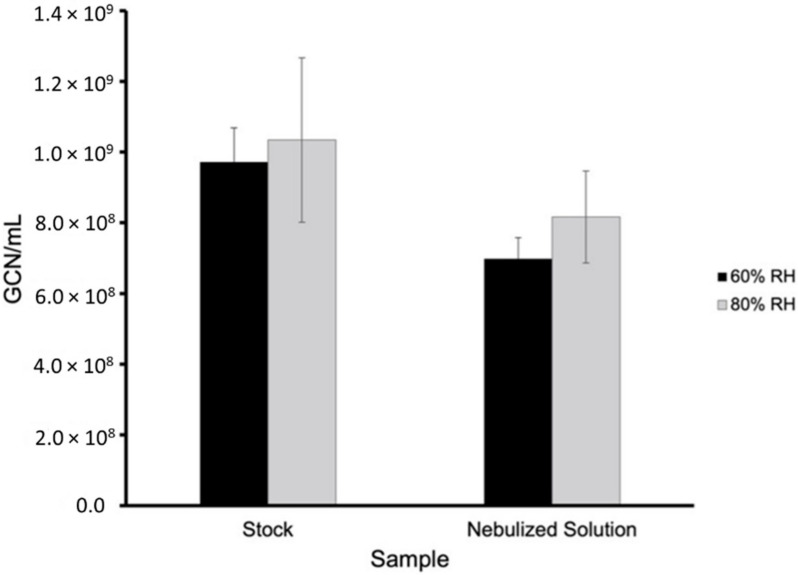
GCN/mL BcoV at 25 °C and 60% and 80% relative humidity of the stock and nebulized solution samples (Table S2 in Supplementary Material).

**Figure 7:**
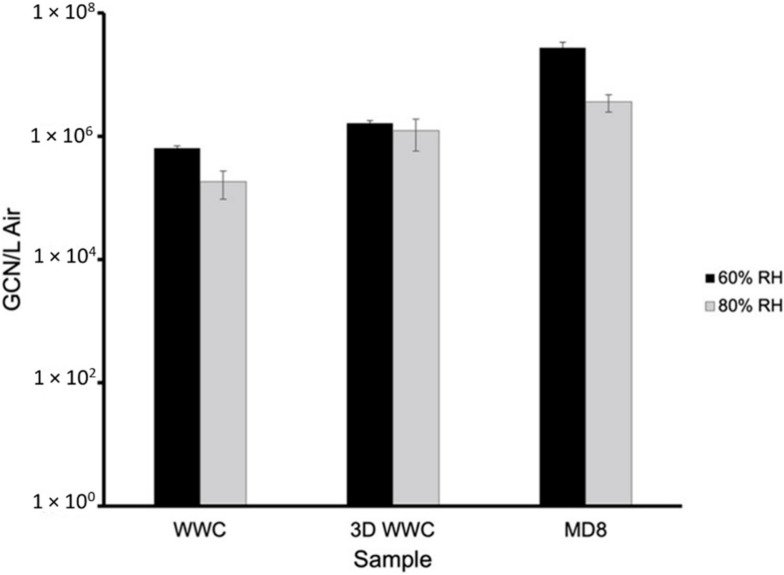
GCN/L Air of BCoV at 25 °C and 60% and 80% relative humidity of aerosol samples collected by the WWC, 3D WWC, and the MD8 sampler (Table S2 in Supplementary Material).

**Figure 8:**
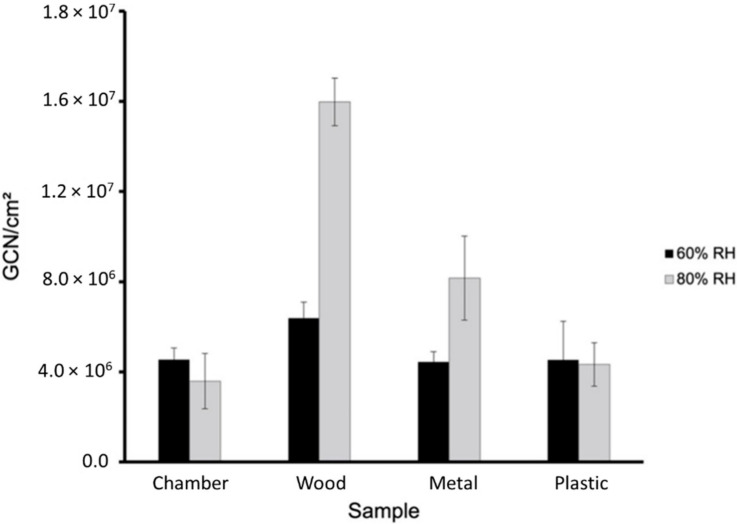
GCN/cm^2^ of BCoV at 25 °C and 60% and 80% relative humidity of the chamber swab, the wood, metal, and plastic surface swab samples (Table S2 in Supplementary Material). The results of BCoV association, Figure 9, and dissociation, Figure 10, to surfaces were obtained using the BLItz system. Figure 9 shows the association constant for wood, metal, and plastic surface swab samples at 60% and 80% relative humidity. The k_a_ values of the wood, metal, and plastic swab samples are 8.733 × 10^6^ Ms^−1^, 6.948 × 10^6^ Ms^−1^, and 9.842 × 10^6^ Ms^−1^ for 60% relative humidity and 7.749 × 10^6^ Ms^−1^, 8.658 × 10^6^ Ms^−1^, and 1.065 × 10^7^ Ms^−1^ for 80% relative humidity, respectively. Figure 10 shows the dissociation constant for wood, metal, and plastic surface swab samples at 60% relative humidity and 80% relative humidity. The k_d_ values of the wood, metal, and plastic swab samples are 1.302 × 10^−4^ s^−1^, 1.00 × 10^−7^ s^−1^, and 1.00 × 10^−7^ s^−1^ for 60% relative humidity and 3.071 × 10^−6^ s^−1^, 1.00 × 10^−7^ s^−1^, and 1.00 × 10^−7^ s^−1^ for 80% relative humidity, respectively. The ANOVA test was performed comparing the different surface swab samples k_a_ values at 60% and 80% relative humidity returned *p*-values of 0.811 and 0.247. There was no significant difference between the k_a_ values of the swab surface samples at 60% or 80% relative humidity. Based on the ANOVA tests performed for each swab sample comparing the 60% and 80% results, there was no significant difference between the k_a_ at 60% and 80% relative humidity. The ANOVA test performed comparing the different surface swab samples at 60% and 80% relative humidity returned *p*-values of 5.25 × 10^−6^ and 0.422. There was significant difference between the k_d_ values of the swab surface samples at 60% relative humidity but not at 80% relative humidity. The wood swab sample was the only surface sample with a significantly different k_d_ value between the 60% and 80% relative humidity tests with a *p*-value of 2.59 × 10^−4^. The swab samples from metal and plastic exhibited stronger association at 80% relative humidity, though not with a significant difference. The swab sample from wood showed stronger dissociation at 80% relative humidity compared to 60%. The difference may be explained by the increased hydrophobicity of the PP (~18 MPa^1/2^) polymer compared to the lower value for PU (~24 MPa^1/2^) based on the Hansen model [36]. Polymers composed of exclusively hydrocarbon constituents (PP) are most hydrophobic, while PU may interact at higher humidity with the phosphate ions in the PBS buffer, resulting in the detachment of the virus particles from the surface. In a recent study with bovine coronavirus, the authors found increased attachment to hydrophobic surfaces [29].

Tests were conducted at 60% and 80% relative humidity at room temperature. Figure 6 shows the total gene copy numbers (GCN) of BCoV per milliliter (mL) in the 60% and 80% relative humidity testing of the stock and nebulized solution samples quantitated by real-time qPCR testing. The total GCN/mL in the stock and nebulized solution were 9.720 × 10^8^ and 6.979 × 10^8^ for 60% relative humidity and 1.034 × 10^9^ and 8.162 × 10^8^ for 80% relative humidity, respectively. Figure 7 and Figure 8 show the total BCoV gene copy numbers (GCN) per liter air (L Air) for the aerosol samples collected by the two wetted wall cyclones and the MD8 sampler, and the total BCoV gene copy numbers per cm^2^ for the chamber and surface swab samples in the 60% and 80% relative humidity testing. The total GCN/L Air for the WWC, 3-D WWC, and MD8 air samplers were 6.432 × 10^5^, 1.645 × 10^6^, and 2.731 × 10^7^ for 60% relative humidity and 1.841 × 10^5^, 1.239 × 10^6^, and 3.618 × 10^6^ for 80% relative humidity, respectively. The total GCN/cm^2^ for the chamber, wood, metal, and plastic surface swab samples were 4.547 × 10^6^, 6.377 × 10^6^, 4.444 × 10^6^, and 4.534 × 10^6^ for 60% relative humidity and 3.590 × 10^6^, 1.597 × 10^7^, 8.162 × 10^6^, and 4.329 × 10^6^ for 80% relative humidity, respectively. Based on statistical analysis performed, only the stock solution sample had significant difference between replicates with a *p*-value of 0.0448. Based on the results from the ANOVA test, all samples had significant difference between replicates with each *p*-value being below 0.05. Higher counts of BCoV were found in the 60% relative humidity testing samples compared to the 80% relative humidity testing samples. The WWC and MD8 Sartorius aerosol samples were the only sets to have significantly different total BcoV gene copy numbers per sample collected. The *p*-value attained from the ANOVA test between the 60% and 80% relative humidity tests for the WWC and MD8 samplers were 0.0147 and 9.30 × 10^−5^. The chamber and wood swab samples had *p*-values of 0.0469 and 0.0134, showing significant difference between the samples at 60% and 80% relative humidity. At 80% relative humidity there was significant difference between the chamber, plastic, metal, and wood samples as the ANOVA test returned a *p*-value of 0.0007.

### 3.2. Hospital Model Room

**Figure 11:**
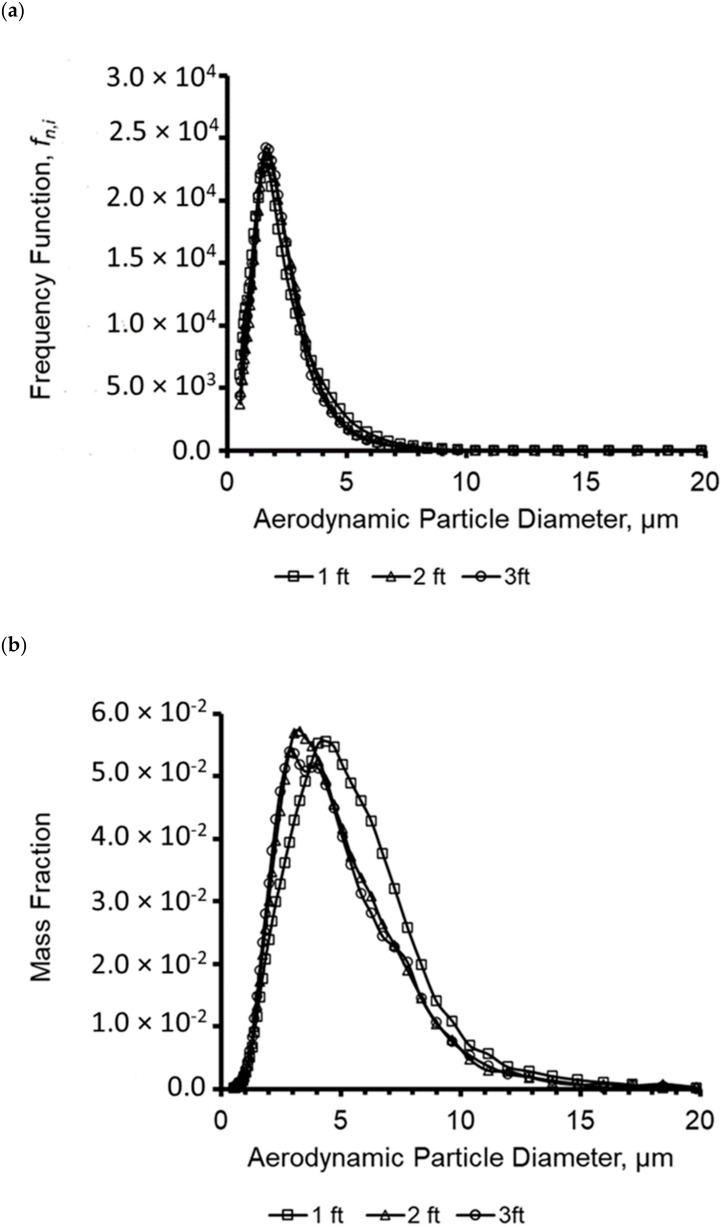
(**a**) Particle size distribution and (**b**) mass distribution of the aerosols produced by the nebulization of the virus suspension. The particle collection recorded analyzed 30.48 cm (head of the bed), 60.96 cm (middle of the bed), and 91.44 cm.

Aerosol particle size distribution and mass concentration in the hospital model room were analyzed at 30.48 cm (head of the bed), 60.96 cm (middle of the bed) and 91.44 cm (foot of the bed) distance from the nebulizer. Figure 11 shows that the number median aerodynamic diameter (NMAD) was approximately 1.6 µm, 1.72 µm, and 1.6 µm. The mass median diameter was 4.07 µm, 3.52 µm, and 3.52 µm, respectively. Yang et al., found that the average size distribution for coughed droplets was in the range of 0.62 µm–15.9 µm with an increased frequency at 1 µm, 2 µm, and 8 µm, further validating the results presented in Figure 11 showing an increased frequency for particle sizes between 1 µm and 2 µm [37].

**Figure 12:**
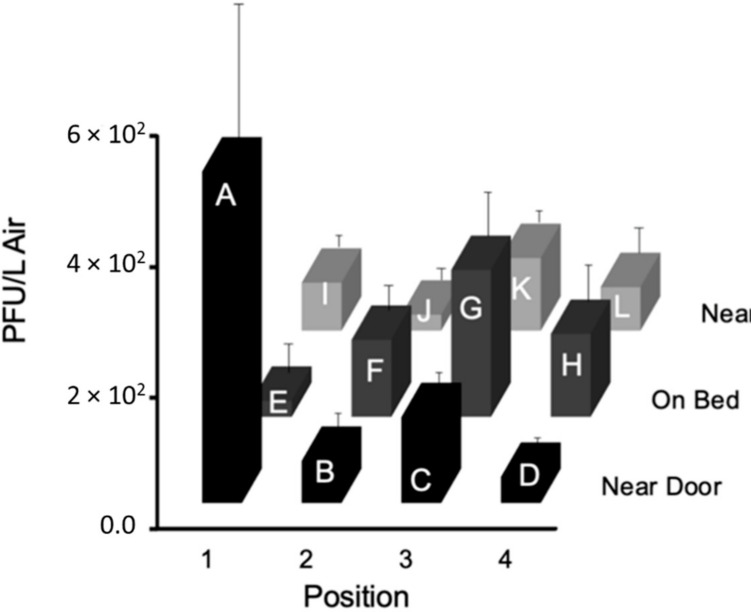
The average PFU/L Air for each PM2.5 sampler at the twelve locations A, B, C, D, E, F, G, H, I, J, and K in the ¾ scale hospital model room using configuration ‘a’ (Table S5 in Supplementary Material).

Using configuration ‘a’ in Figure 5, the particles were more heavily concentrated around the door and the exhaust. The first test of the three replicates resulted in 1333 PFU/L Air phage concentration at Sampler A, which was higher than the collections of the other samplers; however, there was no significant difference between the tests according to the *p*-value, 0.5419, obtained from ANOVA. The second test showed the highest concentration of particle collection at Sampler G, 620 PFU/L Air. Both test 1 and 2 resulted in high particle collection at the door and on the bed. Test 3 had high particle collection at the door, near the exhaust, and at the foot of the bed with the highest particle collection at Sampler K, 273 PFU/L Air. Figure 12 shows the average particle flow based on the three tests.

**Figure 13:**
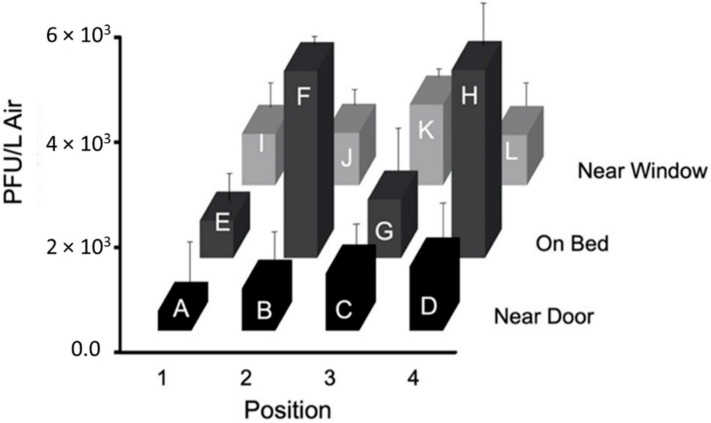
The average PFU/L Air for each PM2.5 sampler at the twelve locations A, B, C, D, E, F, G, H, I, J, and K in the ¾ scale hospital model room using configuration ‘b’ (Table S6 in Supplementary Material).

Results from the tests using configuration ‘b’, without an air curtain, are shown in Figure 13. The first test had the highest PFU/L Air at the foot of the bed (Sampler H, 8833 PFU/L Air), with the second highest at the patient’s head (Sampler F, 4667 PFU/L Air). Sampler F consistently collected high numbers of PFU/L Air during all three tests. Figure 13 shows the average particle flow based on the three tests conducted using this configuration, with the highest average number of PFU/L Air collected at the bed (Samplers F and H, 3556 PFU/L Air and 3578 PFU/L Air). For this configuration, particles are more heavily concentrated at the patient’s head, Sampler F, and feet, Sampler H. Based on the *p*-values obtained from ANOVA analysis, there was no significant difference between the sample PFU/L Air from test to test. The samples from each test were similar and the standard deviation between the average and each test sample at sampler location is not statistically significant.

**Figure 14:**
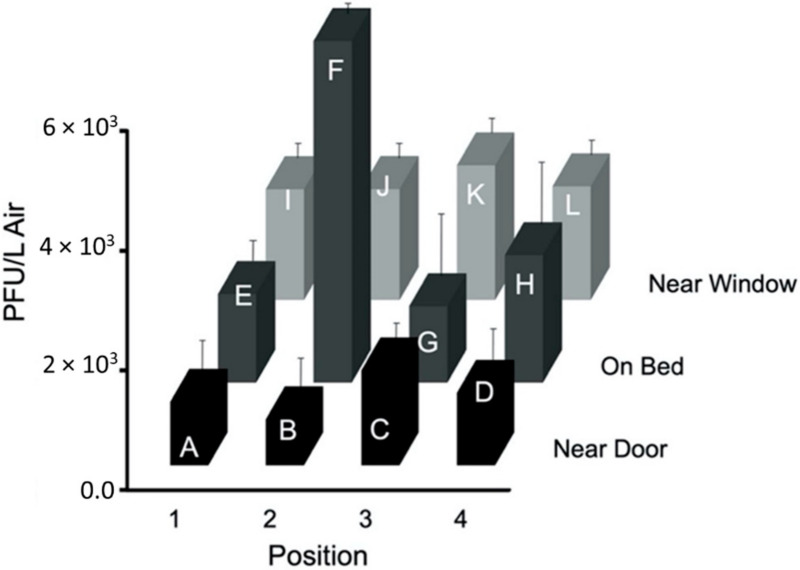
The average PFU/L Air for each PM2.5 sampler at the twelve locations A, B, C, D, E, F, G, H, I, J, and K in the ¾ scale hospital model room using configuration ‘c’ (Table S7 in Supplementary Material).

The results from tests using configuration ‘c’, with an air curtain, are shown in Figure 14. The first test had the highest phage concentration at Sampler F, 4347 PFU/L Air. Sampler F consistently collected high virus numbers of during all three tests. Figure 14 shows the average particle flow based on the three tests conducted using this configuration, with the highest average phage number collected at Sampler F, 5600 PFU/L Air. For this configuration, particles were concentrated at the patient’s head. The *p*-values obtained from ANOVA analysis suggested that there was no significant statistical difference between the three tests performed.

**Figure 15:**
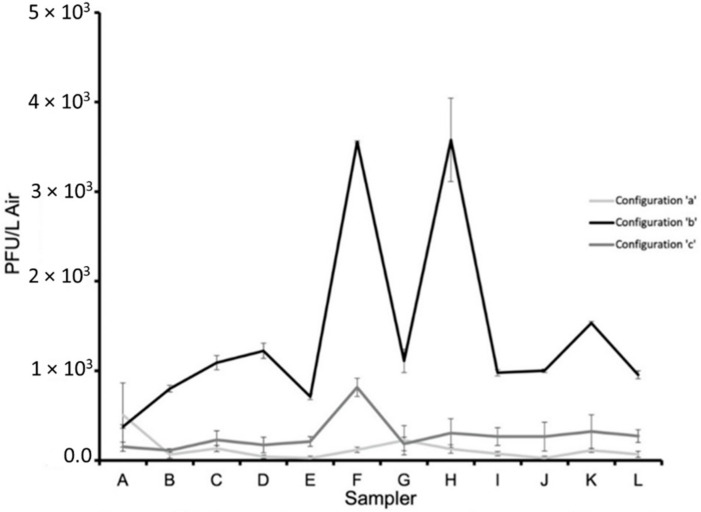
The average PFU/L Air at each sampler at the twelve locations A, B, C, D, E, F, G, H, I, J, and K for each of the configurations, a, b, and c. All PFU/L Air counts were normalized based on the stock PFU counts from the configuration ‘b’ testing (Table S8 in Supplementary Material).

Figure 15 shows the average PFU/L Air graphs for all configurations normalized to the stock PFU counts of configuration ‘b’. Comparing the results, adding the air curtain decreases the concentration of particles at the patient’s head and throughout the room in comparison to without the air curtain in configuration ‘b’. Configuration ‘a’ decreased the particle concentration at the patient’s head but had the highest concentration at Sampler A by the door in the hospital test room. Based on the *p*-value, 0.832, obtained from the ANOVA test, there is no significant difference between the average results from the three configurations.

### 3.3. Air Flow Modeling

**Figure 16:**
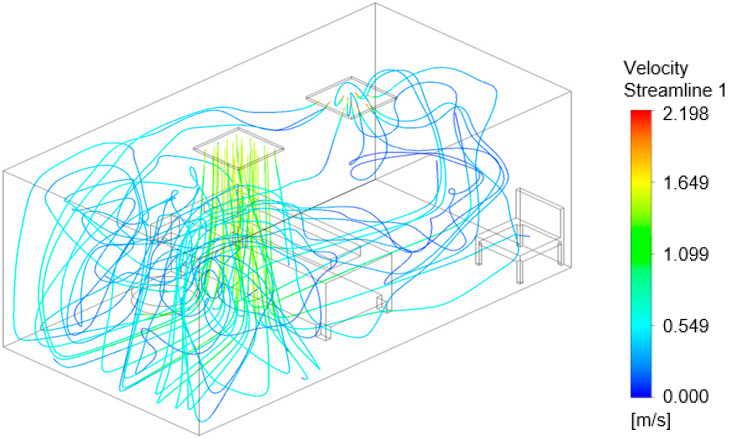
Computational flow model showing airflow velocity streamlines for configuration ‘a’.

**Figure 17:**
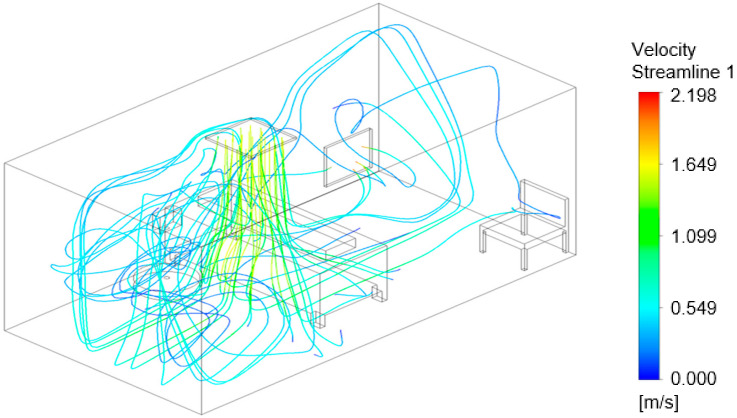
Computational flow model showing airflow velocity streamlines for configuration ‘b’.

**Figure 18:**
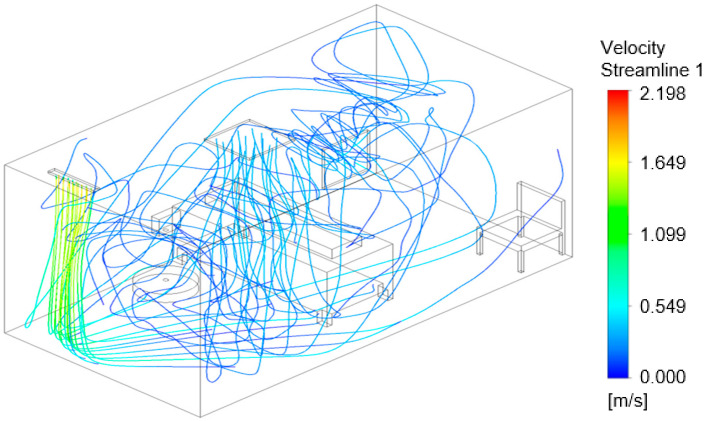
Computational flow model showing airflow velocity streamlines for configuration ‘c’.

The flow models in Figure 16, Figure 17 and Figure 18 show the air velocity streamlines for each configuration. Figure 16, configuration ‘a’, shows many turbulent vortices appearing at the door, potentially resulting in the high pathogen numbers in the air at Sampler A (Figure 12). The model for configuration ‘b’, Figure 17, suggests that the air remains at and moves between the door and the bed with few streamlines going toward the exhaust. The flow model shown in Figure 18 demonstrates that the air curtain is pushing the air velocity streamlines away from the door with more streamlines circulating in the middle of the room and exiting at the exhaust outlet. With configuration “b” and “c”, a greater portion of the air streamlines remains and moves around the bed (Figure 17 and Figure 18), where the nebulizer was at during the experimental setup, which likely resulted in the high pathogen concentration at Samplers F and H as shown in Figure 13 and Figure 14.

## 4. Discussion

Different air inlet and outlet combinations including installation of an air curtain were tested to inhibit the spread of virus. Effects of different ventilation geometries were shown on velocity distribution and distribution of bioaerosols in the room. The experimental data collected in the hospital test room correlate with the results of the flow models. The addition of an air curtain leads to less turbulent air flow at the entrance and a decrease in virus particles remaining in the room. Highest phage concentration was detected at the patient’s head, Sampler F. However, the concentration decreased significantly with the air curtain installed. Stronger coronavirus association was measured on plastic and metal surfaces that are commonly used in hospital rooms and frequently touched by patients and healthcare workers, while stronger dissociation was found from the varnished wood surface at 80% relative humidity. Total counts of BCoV were higher at 60% relative humidity compared to 80%.

The results may help recommend necessary changes in ventilation design to decrease residence time of contaminated air and protect health care workers and visitors. Using the information from the experimental testing and simulated model for the virus particles movement in a hospital room in combination with how virus particles deposit, resuspend, attach, and detach from surfaces when only one variable changes will help delineate factors that might reduce infection and the spread of SARS-CoV-2.

Future work will focus on virus particle tracking and movement under different environmental conditions within the plexiglass chamber. Additional testing will be completed in the hospital test room for longer periods at different air velocities to determine optimal air exchange rates.

## Figures and Tables

**Figure 9:**
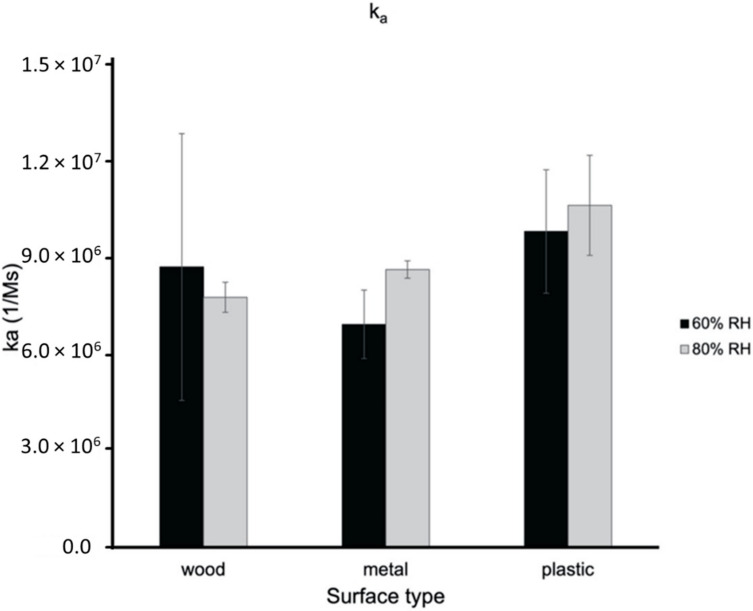
Association constants (k_a_) of wood, metal, and plastic swab surface samples at 60% and 80% relative humidity (Table S3 in Supplementary Material).

**Figure 10:**
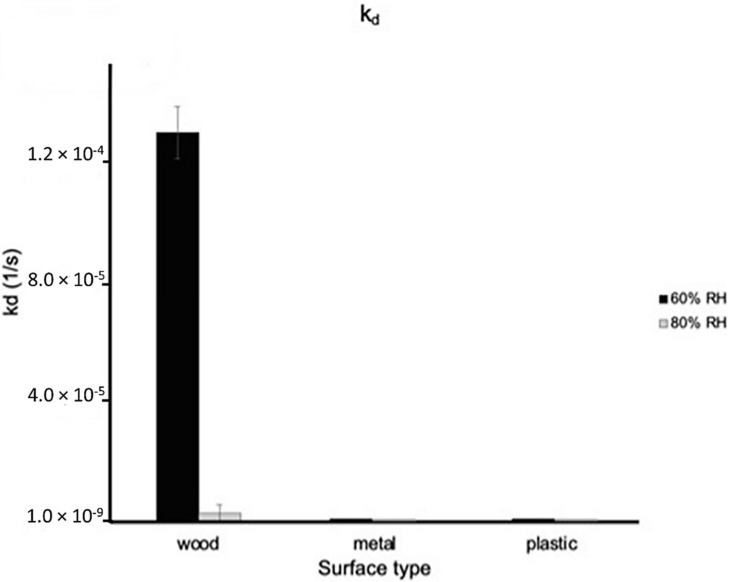
Dissociation constants (k_d_) of wood, metal, and plastic swab surface samples at 60% and 80% relative humidity (Table S3 in Supplementary Material).

**Table 1:** Initial conditions used in ANSYS models.

Temperature	27 °C
Inlet	Velocity inlet (1.5 m/s)
Exhaust	Pressure outlet (0 atm)
Air curtain (config. ‘c’ only)	Velocity inlet (11.5 m/s)
Walls	No slip, stationary
Tolerance (x)	10^−3^
Tolerance (y)	10^−3^
Tolerance (velocity)	10^−3^
Tolerance (k)	10^−3^
Tolerance (ω)	10^−3^
Tolerance (energy)	10^−6^

## Data Availability

All data associated with this manuscript are available in the body of the paper and in the Supplementary material.

## Supplementary Materials

The following are available online at https://www.mdpi.com/article/10.3390/v14030616/s1, Table S1. ANOVA Test *p*-value Data for Section 3.1; Table S2. Figure 6, Figure 7 and Figure 8 Data; Table S3. Figure 9 and Figure 10 Data; Table S4. ANOVA Test *p*-value Data for Section 3.2; Table S5. Figure 12 Data; Table S6. Figure 13 Data; Table S7. Figure 14 Data; and Table S8. Figure 15 Data.
