# Supplementary material for: Environmental Effects on Viable Virus Transport and Resuspension in Ventilation Airflow
Source: Viruses. 2022 Mar 16;14(3):616. doi: 10.3390/v14030616 (PMC8950092; doi:10.3390/v14030616)
Supplement: Supplementary file 1 [file viruses-14-00616-s001.zip › viruses-1536246-supplementary.pdf]

Supplementary Material

Table S1. ANOVA Test p-value Data for Section 3.1

FOR 60% RH

Sample	p-value
WWC	0.17514034
3D WWC	0.06550033
MD8	0.98203972
Stock	0.04478175
Nebulized	0.07697754

FOR 80% RH

Sample	p-value
WWC	0.02429704
3D WWC	0.01386592
MD8	0.00950685
Stock	0.03418927
Nebulized	0.02552379

60% RH vs 80% RH

Sample	p-value
WWC	0.01472075
3C	0.63338629
MD8	9.3036E-05
Stock	0.83208904
Nebulized	0.49565201

ANOVA Between Chamber Replicates

RH %	p-value
60	0.95050882
80	0.86209744

ANOVA Swab Sample

Sample	p-value
Chamber	0.04688922
Plastic	0.84952074
Metal	0.16835071
Wood	0.01338988

ANOVA Between Samples

RH %	p-value
60	0.59981991
80	0.00074043

Comparing Swab Samples ka

RH	p-value
60%	0.81099178
80%	0.24703956

Comparing Swab Samples kd

RH	p-value
60%	5.2504E-06
80%	0.421875

For ka

Sample	p-value
wood	0.85533566
metal	0.24984041
plastic	0.79006595

For kd

Sample	p-value
wood	0.00025924
metal	no variance
plastic	no variance

Table S2. Figure 6, 7, and 8 Data

60% Relative Humidity

Sample Names	GCN	Units	Standard Deviation
Stock	972012107	GCN/mL	193134713.1
Nebulized Solution	697977041	GCN/mL	119664749.3
WWC	643259.496	GCN/L Air	115406.777
3D WWC	1645871.96	GCN/L Air	311698.6399
MD8	27316040.2	GCN/L Air	12440760.96
Chamber	4547789.045	GCN/cm^2	1023406
Wood	6377732.571	GCN/cm^3	1443652
Metal	4444785.993	GCN/cm^4	900630.7
Plastic	4534921.556	GCN/cm^5	3423972

80% Relative Humidity

Sample Names	GCN	Units	Standard Deviation
Stock	1034229236	GCN/mL	4.65E+08
Nebulized Solution	816214654.5	GCN/mL	2.61E+08
WWC	184119.3867	GCN/L Air	176779.4
3D WWC	1239223.074	GCN/L Air	1329106
MD8	3618324.796	GCN/L Air	2283433
Chamber	3590322	GCN/cm^2	2451593.1
Wood	15975990	GCN/cm^3	2115857.8
Metal	8162300	GCN/cm^4	3726511.41
Plastic	4329449	GCN/cm^5	1932944.07

Table S3. Figure 9 and 10 Data

Sample	ka	Standard Deviation
wood 80RH	7794000	465246.977
metal 80RH	8658666.67	270754.655
plastic 80RH	10655333.3	1559075.47
wood 60RH	8733000	4157134.74
metal 60RH	6948666.67	1067286.32
plastic 60RH	9842666.67	1919350.24

Sample	kd	Standard Deviation
wood 80RH	3.071E-06	2.573E-06
metal 80RH	0.0000001	0
plastic 80RH	0.0000001	0
wood 60RH	0.00013027	8.6558E-06
metal 60RH	0.0000001	0
plastic 60RH	0.0000001	0

Table S4. ANOVA Test p-value Data for Section 3.2

Configuration	p-value
a	0.54190119
b	0.29419591
c	0.06323661

Configuration Comparison p-value	
p-value	0.83229616

Table S5. Figure 12 Data

Sample	PFU/L Air	Standard Deviation
A	507	715.9143
B	64	80.09254
C	131	67.65928
D	40	36.71714
E	24	43.71626
F	118	61.10101
G	224	327.301
H	127	94.59465
I	73	46.66667
J	24	47.29733
K	111	43.37605
L	67	68.42135

Table S6. Figure 13 Data

Sample	PFU/L Air	Standard Deviation
A	378	19.24501
B	800	37.11843
C	1089	78.97491
D	1222	85.72134
E	711	31.50544
F	3556	10.1835
G	1111	129.3288
H	3578	465.8246
I	978	33.55482
J	1000	20
K	1533	17.63834
L	956	44.38885

Table S7. Figure 14 Data

Sample	PFU/L Air	Standard Deviation
A	1060	722
B	769	268
C	1587	1427
D	1211	1219
E	1480	767
F	5707	1416
G	1276	1081
H	2129	2246
I	1851	1404
J	1853	2252
K	2249	2620
L	1898	994

Table S8. Figure 15 Data

Sample	Configuration 'a'	Configuration 'b'	Configuration 'c'
A	507	378	151
B	64	800	110
C	131	1089	227
D	40	1222	173
E	24	711	211
F	118	3556	815
G	224	1111	182
H	127	3578	304
I	73	978	264
J	24	1000	265
K	111	1533	321
L	67	956	271
